# Gunshot Injury to the Face With Atypical Non-linear Bullet Trajectory: A Case Report

**DOI:** 10.7759/cureus.43917

**Published:** 2023-08-22

**Authors:** Nilabh P Singh, Ravi Kumar Sharma, Amit Patil

**Affiliations:** 1 Forensic Medicine and Toxicology, All India Institute of Medical Sciences, Raipur, Raipur, IND; 2 Forensic Medicine, All India Institute of Medical Sciences, Patna, Patna, IND; 3 Forensic Medicine and Toxicology, All India Institute of Medical Sciences, Patna, Patna, IND

**Keywords:** head and neck, wound ballistics, non-linear trajectory, firearm injury, bullet

## Abstract

The disruptive effect of firearm missiles on body tissues depends on many factors. However, it mainly relates to the bullet's physical and dynamic properties and tissue-related factors. We encountered an unusual case of a gunshot injury wherein the bullet traversed the neck with an upward and non-linear trajectory with an exit from the contralateral side of the neck without damaging any vital neck organs. A 26-year-old male presented with a gunshot wound to the chin from close range. A bullet entry hole was observed on the right side of the chin, encircled by the abrasion collar, with tattoo marks around the area. The patient was conscious, with normal vital signs and no injury to the cranial nerves or aerodigestive tract. The CT imaging of the patient revealed the injury tract traversing through the muscles of the floor of the mouth to involve the left carotid and left parapharyngeal space, along with the left sternocleidomastoid muscle, with an exit hole below the left mastoid in the posterior triangle of the neck. A bullet usually travels through the body in a straight line or pathway; however, its non-linear trajectories depend on the projectile's dynamics and its interaction with the body tissues. The present case emphasizes understanding wound ballistics to know the erratic bullet trajectories in the victim's body and their interpretation, irrespective of their entry site.

## Introduction

Examining firearm injuries and their interpretation is routine forensic casework for the deceased and living victims within the scope of forensic pathology and clinical forensic medicine. Gunshot head, face, and neck injuries carry high morbidity and mortality rates by damaging vital and essential structures, including the central nervous system, jugular veins, and carotid arteries [1]. The way they are sustained or inflicted ranges from homicide to accident; the rates, however, vary depending on the region and the country [2]. In 2019, a worldwide report estimated that of the total 250,227 firearm-related fatalities, 65.9% occurred in six countries: Brazil, the United States, Venezuela, Mexico, India, and Columbia. India was ranked fifth in the chart with a total estimated death toll of 14,710 [3]. Despite stringent gun laws in India that do not allow civilians or any person to use guns for any reason, various country-made handguns, or "desi katta," are available for somewhere between Rs 2,500 and Rs 15,000, and different seized illegal firearms from illegal factories show the extent of the burden of the problem [4].

Firearm injuries are complicated and aggressive traumatic injuries that are challenging to many surgeons and clinicians, especially in their management. More so in forensic settings, the projectile trajectory and the firearm shot ranges are crucial aspects that must be determined while reconstructing a gun shooting incident. Usually, a bullet with perforating injuries follows a straight path through the body from the entry wound to the exit wound. But large deflections in the trajectory are possible when deflected by intermediate tissues. Still, as reported, a few patients with head and neck firearm injuries have survived without lethal organ damage due to a non-linear course [5,6]. Here, we present a highly unusual case of gunshot injury crossing the neck, wherein the bullet entered from the chin got deflected from the mandibular bone and travelled non-linearly and upward through the structures of the neck, exiting from the contralateral side of the neck just below the mastoid region.

## Case presentation

A 26-year-old male riding a motorcycle was shot by an assailant from close range in a robbery attempt. The victim fell, became unconscious, and was brought to a nearby primary healthcare hospital by a passerby, who admitted him. The victim gained consciousness, was treated primarily by cleaning and dressing the wound, and was then referred to our hospital for further evaluation and management.

**Figure 1:**
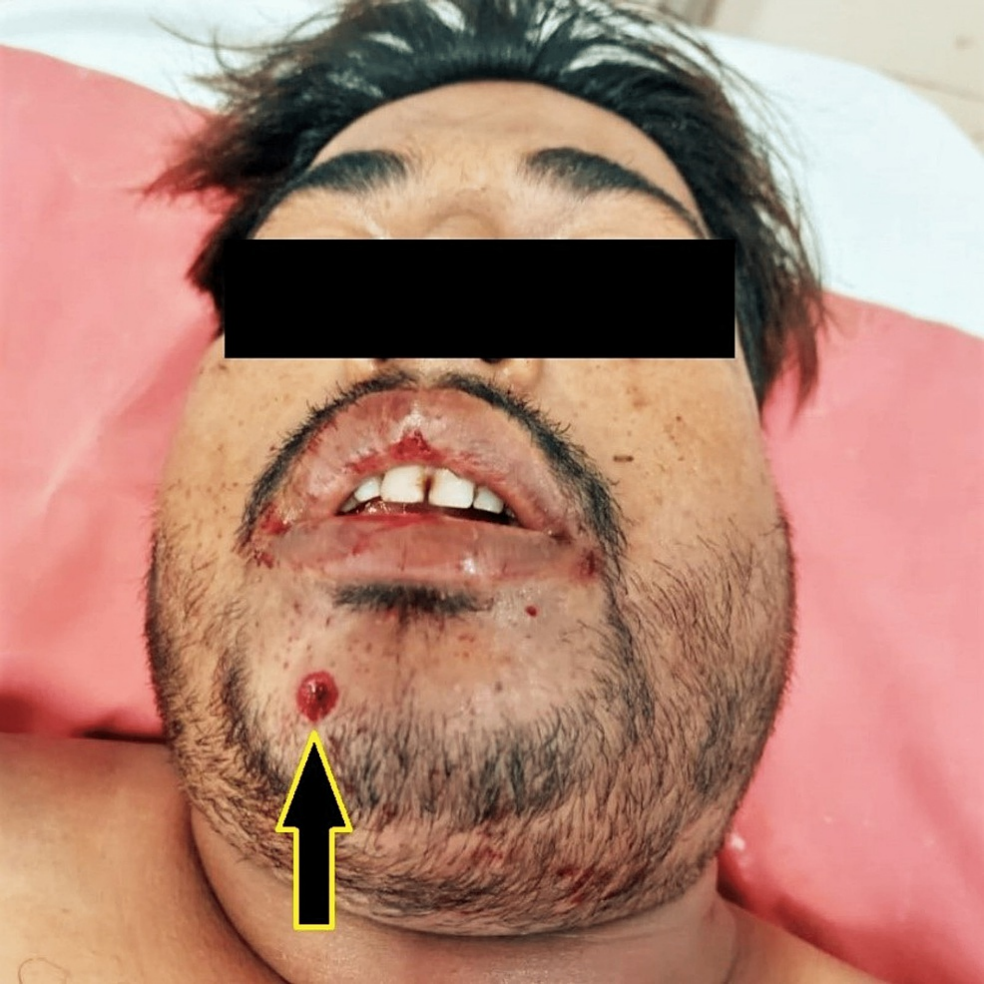
Bullet entry wound over the right side of the chin with powder tattooing.

**Figure 2:**
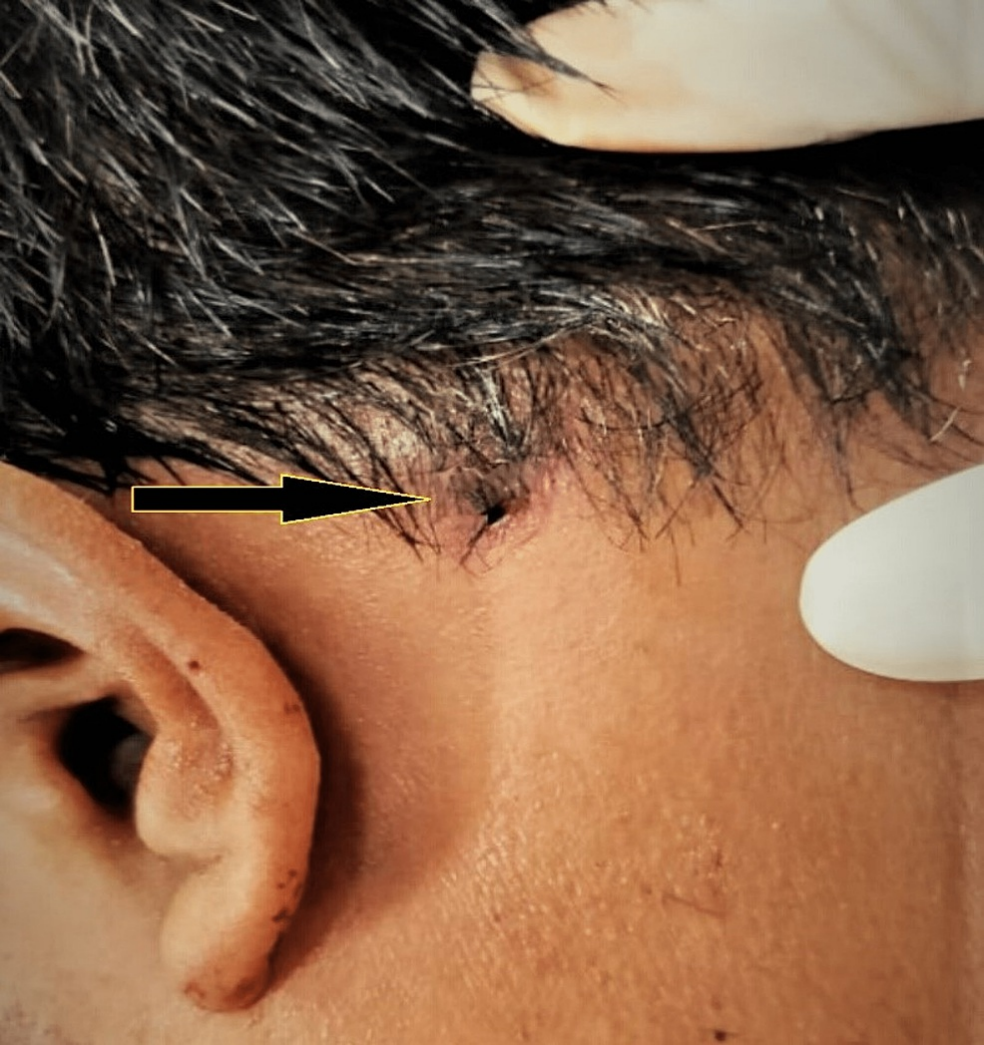
Exit wound at the left side of the neck below the mastoid.

On admission, the victim was conscious and oriented with a Glasgow Coma Scale (GCS) rating of 15, his BP was 134/80 mm of Hg, and his pulse was 110 beats/minute. The victim had difficulty vocalisation because of the pain and complained of poor oral bleeding. Forensic examination of the injury revealed a firearm entry wound on the right side of the chin, 0.5 cm in diameter, 1.5 cm below the lip, and 1 cm right to the midline, encircled by the abrasion collar, bright red (Figure 1). Multiple pinhead-sized tattoo burn marks surrounded the entry wound in an area of 6 cm × 8 cm. The bullet entered the oral cavity by fracturing the mandibular body on the right side; by visual examination, the mandible was seen to be divided into two pieces between the right lower central and lateral incisors. Further, it travelled below the tongue and the soft tissues around it towards the left and backward, exiting from the neck from the left side just below the mastoid region. The exit wound was irregular, 1 cm × 0.5 cm, at the left lateral upper neck, 1 cm below the left mastoid, and 10 cm left to the midline, bright red in colour (Figure 2). The direction of the shot was from forward, downward, and right to backward, upward, and left.

**Figure 3:**
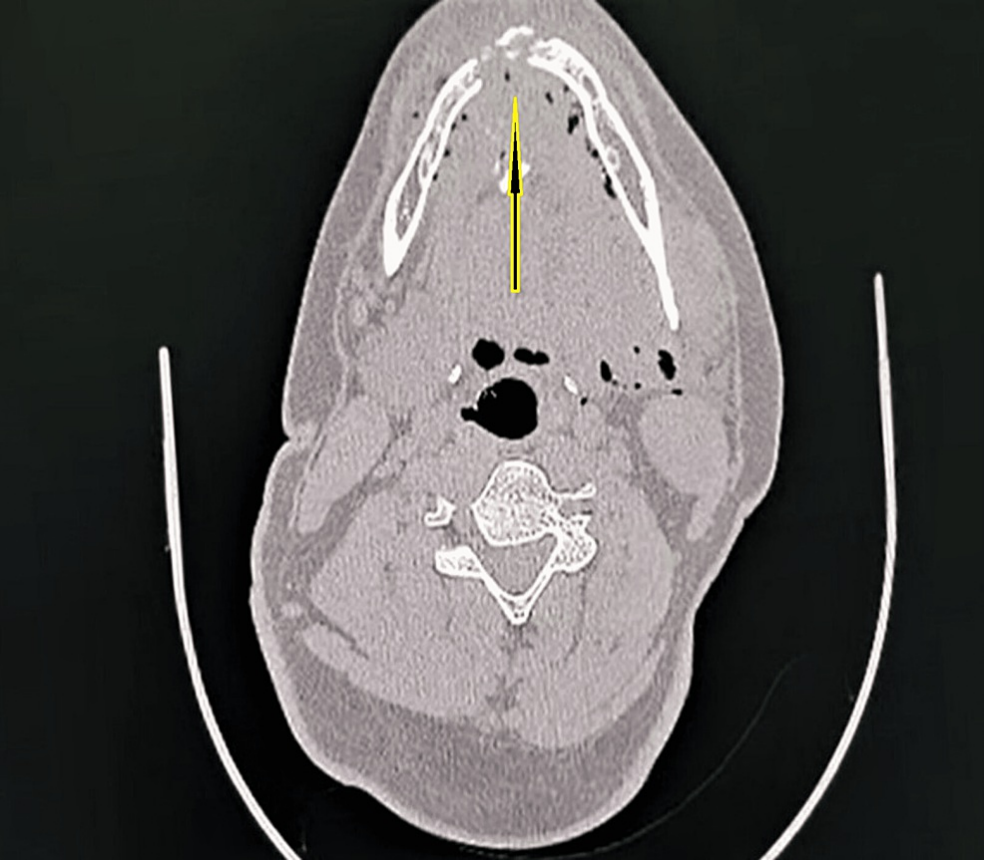
Non-contrast-enhanced CT image of the head showing the base or floor of the mouth with fracture of the mandible and air foci in the soft tissues of the mouth.

**Figure 4:**
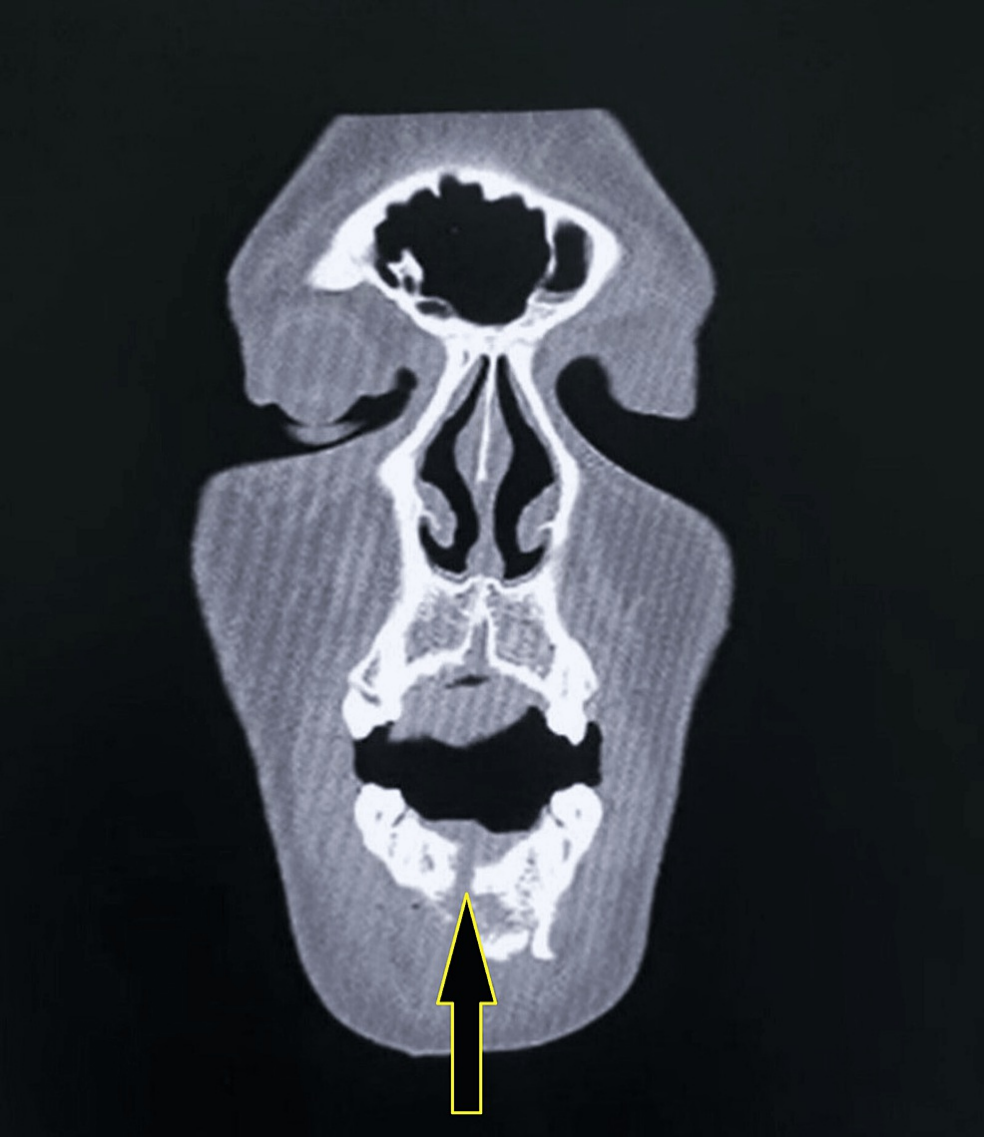
Non-contrast-enhanced CT image of the head showing the base or floor of the mouth with fracture of the mandible and air foci in the soft tissues of the mouth.

**Figure 5:**
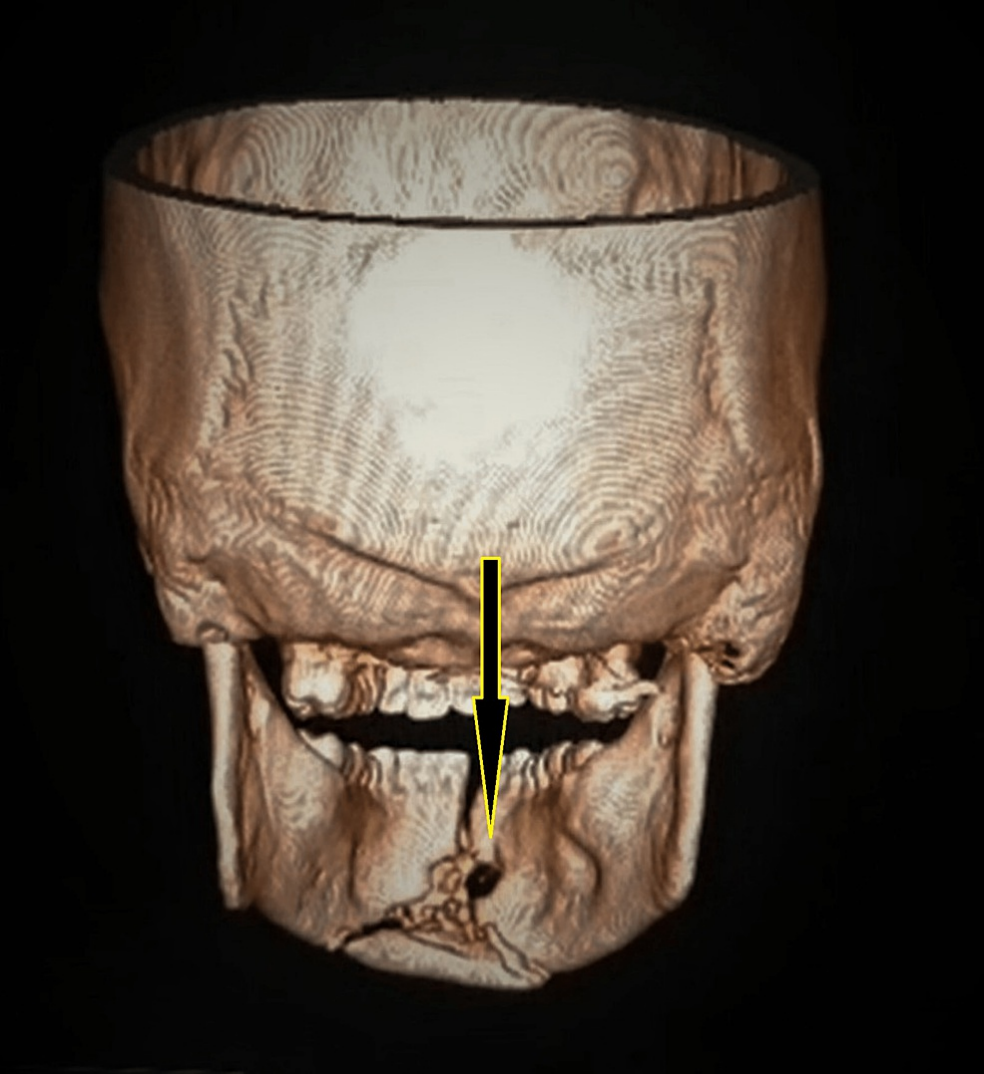
Non-contrast-enhanced CT three-dimensional reconstruction revealed three significantly large, fractured segments of the mandible.

The patient was managed conservatively after the initial assessment and sent for non-contrast computed tomography (NCCT) of the head and face on the day of admission. The NCCT head revealed no abnormality; however, the NCCT of the face report stated, "the entry wound is noted at the oral cavity, with the injury tract traversing through the muscles of the floor of the mouth and extending posteriorly to involve the left carotid and left parapharyngeal space along with the left sternocleidomastoid muscle, with the possible exit wound lying inferolateral to the left mastoid in the posterior triangle of the neck. A comminuted fracture in the symphyseal region of the mandible involving the alveolar process was present. Air foci with a few bone fragments were seen throughout the trajectory along the floor of the mouth. A linear undisplaced fracture of the left mandibular neck is seen with inferomedial dislocation of the bilateral temporomandibular joint" (Figures 3-4). The CT angiography done for the neck and head structure on the third day of the incident revealed no abnormality. NCCT three-dimensional reconstruction revealed three significantly large, fractured segments of the mandible (Figure 5).

On the 12th day of the incident, the oral and maxillofacial surgery department managed the patient surgically by ‘open reduction and internal fixation’ under general anaesthesia by fixing the fragmented mandibular parts with bars and plates, and the procedure was uneventful.

## Discussion

The main reason for high mortality after a penetrating injury to the neck is massive haemorrhage due to a significant vascular injury [7]. The head and neck region are compactly packed with vital anatomical neurovascular structures such that a minimal penetrating injury can impose fatal damage on them, with high mortality rates associated with head and neck-related firearm injuries in the range of 35-36% [8,9].

Firearm-related injuries are affected by many factors but are primarily related to the projectile and tissue properties [10,11]. The projectile-related elements consist of the physical properties of the bullet, such as its mass, velocity, calibre, shape, trajectory, spinning motion, distance travelled by the bullet, and the type and calibre of the barrel [10,11]. Amongst all these, the wounding power of a bullet is determined by the mass and velocity of the fired bullet responsible for the kinetic energy generated (KE = 1/2 mv^2^). The faster a bullet travels, the more kinetic energy (KE) is generated, increasing the potential for tissue damage. Firearm wounds are the direct and indirect effects of the bullet on living tissues. Direct injury, also referred to as "prompt damage," is the result of the fast distension and rupture of the tissue due to the bullet passing through it [10], creating a cavity along the bullet’s track due to the tissue disruption [10,11]. The resultant indirect damage to the tissues develops due to the "temporary cavitation" and "shock wave" (also known as "sonic pressure wave") generated due to the negative pressure around the bullet track and from the maximum pressure point at the leading edge of the bullet [10,11]. The bullets with higher velocity cause more tissue damage through an indirect effect by producing superior pressure changes [10].

In the present case, the bullet entered the body by penetrating the chin from the right side, causing a fracture of the symphysis menti, crossing the neck structures along the floor of the mouth transversely, and producing an undisplaced fracture of the left mandibular neck, thereby exiting from the upper part of the neck. Our victim was fortunate enough to survive the firearm injury by avoiding injury to any vital neck structures, such as the jugular veins, carotid arteries, pharynx, larynx, and cervical spine, though the bullet had almost transversely passed through the neck structures to the opposite side.

As per the history available from the records, the victim was riding on a motorcycle and was shot by the assailant in the same sitting body position from the right side, possibly with the firing arm of the assailant at the level of the victim's face or probably at a little lower level. Ideally, the bullet should have travelled in a straight, linear fashion, entering the body after fracturing the right side of the mandibular body, and could have exited the body through an exit wound on the right side posteriorly after damaging the oral, neck, or cervical vertebral spine. However, in the present case, after entering from the right side of the chin and fracturing the mandible, the bullet got deflected and changed its course towards the left side, thereby exiting from the inferomedial aspect of the left mastoid. In other words, instead of travelling in a straight trajectory with entry and exit both on the right side, it travelled in a non-linear zig-zag path, avoiding damage to the vital neck structures, with direct damage seemingly limited to the fracture of the mandible on the bilateral side and soft tissue injury to the floor of the mouth.

In the absence of projectile particles in the track of the wound and bullet (exited), the history given by the victim (conscious with a GCS score of 15), and the examination of the injury (presence of an abrasion collar and powder tattooing), it is confirmed that the injury was due to a rifled firearm.

A bullet travelling with its "nose on" along its longitudinal axis tends to deviate from its path or trajectory, a phenomenon known as "yawing," and its complete rollover beyond 90 degrees is referred to as "tumbling." The destabilising effect of a yawing or tumbling bullet is counteracted by a high-frequency spinning motion of the bullet around its longitudinal axis, providing gyroscopic stability and maintaining its nose-on orientation. Once a bullet enters the body, it cannot hold its gyroscopic steadiness as the spinning effects are overcome by the tissue density, which is far higher than the air [10]. After entering the body, the bullet may deflect from its path after hitting a bone, deviate entirely from its original trajectory, and create a new track. According to the CT images of our patient, the bullet, after hitting and fracturing the mandibular body, got deflected from its path and then travelled along the floor of the mouth upward towards the left side, subsequently exiting through the inferior portion of the left mastoid.

A bullet is usually assumed to pass through the body in a straight line or pathway. However, the dynamics of the bullet, along with its interaction with body tissues, can explain the non-linear trajectories in the body. In this case, the bullet got deflected upon its impact with the mandible bone, where it lost its most energy in fracturing the mandible and changed its path with an upward and lateral trajectory at the exit, evading all important vital structures of the neck. In this case, the bullet trajectory was non-linear as per the positional relationship between the entry and exit sites. The trajectory of the bullet is non-linear as the track was not directly extending from the entry to the exit wound on the right side, but rather the bullet travelled in a zigzag manner, first from the right side entry wound, causing fracture of the body of the mandible, then travelled along the floor of the mouth, extending posteriorly and upwardly along the left parapharyngeal space, causing an undisplaced fracture of the left mandibular neck, and then exiting inferomedially of the left mastoid region. It is a well-known fact that the extent of tissue damage is directly affected by the deformation and fragmentation of the bullet, which in turn depend primarily on the bullet's design. With their tips exposed from the jacket, the non-jacketed and semi-jacketed bullets can get easily deformed and fragment upon impact, with a more efficient transfer of kinetic energy over a greater target area [10,11]. Unfortunately, the bullet exited in our case and could not be retrieved to know whether it was jacketed.

In perforating bullet injuries, the bullet follows a linear trajectory that can be determined by simple trigonometric measurements of the specific locations of the entry and exit wounds on the body. However, significant deviations in the wound trajectory are possible if the projectile glances or deflects from an anatomical structure such as a bone, tooth, etc., and the magnitude of such deflections or deviations is influenced by various projectile and tissue-related factors such as the physical properties of the missile, its velocity and energy, the nature of the tissues from which the bullet gets deflected, etc. [12-14]. Although many cases have been reported of patients who have survived atypical head and neck gunshot injuries without fatal organ damage [15-17], only a few are reported on non-linear bullet trajectories [5,6]. In addition, a few cases have also been reported of the unexpected route travelled by a bullet that entered from the face into the gastrointestinal (GI) tract after involuntary swallowing [15-17].

## Conclusions

Understanding wound ballistics is of great help in anticipating the amount of tissue injury to the internal organs, the residual effect of damage to the internal structures, the bullet trajectory, and its interpretation. The present case strongly suggests the importance of the erratic nature of a bullet in the victim’s body, which got deflected at the entry from the mandibular bone and travelled in a non-linear fashion, creating an atypical wound trajectory.
